# Physician Attitudes and Confidence Toward Dementia Capability: Screening, Diagnoses, and Referrals

**DOI:** 10.1093/geroni/igaa057.654

**Published:** 2020-12-16

**Authors:** Tamara Herrick, Michelle Ward

**Affiliations:** 1 Mainehealth, portland, Maine, United States; 2 UMAINE, Portland, Maine, United States

## Abstract

The MaineHealth Alzheimer’s Disease Partnership is working to improve integration between the healthcare system and community partners through training and a referral network. Primary care providers are often the first to assess cognitively impaired patients, so it is important to understand their attitudes and confidence in dealing with dementia. The objective of this study is to determine barriers to care and evaluate healthcare providers’ attitudes towards their dementia capability, which includes screening for cognitive impairment, disclosing diagnoses, and making referrals to community-based organizations or specialists. A 27-item survey was developed and sent to 474 providers from MaineHealth practices via email. Fifty-three providers responded to the survey. Five healthcare professionals also took part in a focus group; looking more specifically at challenges encountered throughout the dementia care system. This poster will present the findings from the survey and focus group. There was strong agreement that much can be done to improve the quality of life for patients with dementia (86% agreed/strongly agreed) and that screening all patients over age 65 is important (85% agreed/strongly agreed). Confidence levels in ability to diagnose dementia, provide memory loss information, and refer patients to specialists were significantly associated with training (p<.05). The majority of providers identified barriers to cognitive screening and referring patients to community-based organizations, showing that improvements are needed at the system level to remove these barriers. Overall, the results suggest that dementia specific training can improve confidence in care and allow physicians to provide more information about memory loss to patients.

